# Classifying Different Emotional States by Means of EEG-Based Functional Connectivity Patterns

**DOI:** 10.1371/journal.pone.0095415

**Published:** 2014-04-17

**Authors:** You-Yun Lee, Shulan Hsieh

**Affiliations:** 1 Department of Psychology, National Cheng Kung University, Tainan, Taiwan; 2 Institute of Allied Health Sciences, National Cheng Kung University, Tainan, Taiwan; Brain and Spine Institute (ICM), France

## Abstract

This study aimed to classify different emotional states by means of EEG-based functional connectivity patterns. Forty young participants viewed film clips that evoked the following emotional states: neutral, positive, or negative. Three connectivity indices, including correlation, coherence, and phase synchronization, were used to estimate brain functional connectivity in EEG signals. Following each film clip, participants were asked to report on their subjective affect. The results indicated that the EEG-based functional connectivity change was significantly different among emotional states. Furthermore, the connectivity pattern was detected by pattern classification analysis using Quadratic Discriminant Analysis. The results indicated that the classification rate was better than chance. We conclude that estimating EEG-based functional connectivity provides a useful tool for studying the relationship between brain activity and emotional states.

## Introduction

The question of whether different emotional states are associated with specific patterns of physiological response has long captivated emotion research [1]–[3]. Although some evidence for autonomic (i.e., peripheral physiological response) specificity has been reported [4]–[7], many other studies have indicated that the physiological correlates of emotions are likely to be found in the central nervous system (CNS) rather than simply in peripheral physiological responses [8]–[10]. Researchers have supported this viewpoint using electroencephalographic (EEG) or other neuroimaging (e.g., functional Magnetic Resonance Imaging, fMRI) approaches to investigate the specificity of brain activity associated with different emotional states [11] However, most of the available studies on emotion-specific EEG response have focused on EEG characteristics at the single-electrode level, rather than at the level of EEG-based functional connectivity. Contrary to this trend of single-electrode-level analysis, Mauss and Robinson (2009), in their recent review paper, have indicated that “emotional state is likely to involve circuits rather than any brain region considered in isolation” [11], neuroimaging methods that examine interrelated activity among multiple brain sites may hold more promise for understanding whether and how emotional specificity is instantiated in the brain. In agreement with this view, we believe that analyzing emotional specificity at the level of EEG-based functional connectivity in the brain is a more ecologically valid approach. Therefore, the current study aimed to elucidate whether emotional specificity can indeed be better characterized through EEG-based functional connectivity, using the evaluation criterion of whether the latter serves as a better predictor for recognizing different emotional states.

Earlier EEG-based studies of emotional specificity, with analyses at the single-electrode level, have demonstrated that asymmetric activity at the frontal site (especially in the alpha (8–12 Hz) band) is associated with emotion. For example, Ekman and Davidson (1993) found that voluntary facial expressions of smiles of enjoyment produced higher left frontal activation [12], whereas another study found decreased left frontal activity during the voluntary facial expressions of fear [13]. In addition to alpha band activity, theta band power at the frontal midline (Fm) has also been found to relate to emotional states. For example, Sammler and colleagues proposed that pleasant (as opposed to unpleasant) emotion is associated with an increase in frontal midline theta power [14]. To further demonstrate whether these emotion-specific EEG characteristics, i.e., alpha asymmetry or activity in other frequency bands, are strong enough to differentiate between various emotional states, some studies have utilized a pattern classification analysis approach, and the resulting recognition accuracy has generally been above chance [15]–[18].

Nevertheless, as previously mentioned, emotion is a complex process; hence, examining the issue of EEG-based emotional specificity and the recognition of different emotional states may be more valid if the issue is examined via EEG-based functional connectivity rather than being based simply on analyses at the single-electrode level. There are various ways to estimate EEG-based functional brain connectivity. Correlation, coherence and phase synchronization indices between each pair of EEG electrodes had been used in emotional research. In the early era of EEG research, correlation was most commonly used to investigate the similarity between two EEG signals [19]. Based on the assumption that a higher correlation map indicates a stronger relationship between two signals, correlation has been used in various areas of research, such as the study of sensory stimulation, clinical problems and sleeping [20]. Coherence gives similar information as correlation, but coherence includes the covariation between two signals as a function of frequency, a measure that has been used in many research fields, including physiology [21] *neurological* disorder [22], and exercise physiology [23]. Phase synchronization among the participating neuronal groups is another way to estimate the EEG-based functional connectivity among brain sites; it is estimated based on the phase difference between two signals. Measures of phase synchronization in EEG are usually used in the study of neurological disease [24].

More recently, some researchers have noted the importance of functional brain connectivity in emotion research and have started examining emotional specificity using EEG-based functional brain connectivity; however, most researchers have focused on only one type of connectivity index. For example, Shin and Park (2011) proposed that when emotional states become more negative at high room temperatures, correlation coefficients between the channels in temporal and occipital sites increase more than they do when room temperatures are more moderate [25]. Hinrichs and Machleidt (1992) proposed that coherence decreases in the alpha band during sadness, compared to happiness [26]. Miskovic and Schmidt (2010) found that EEG coherence between the prefrontal cortex and the posterior cortex increases when viewing highly emotionally arousing (i.e., threatening) images, compared to viewing neutral images [27]. Costa and colleagues were the first to apply the synchronization index to detect interaction in different brain sites under different emotional states [28]. Costa et al.’s (2006) results showed an overall increase in the synchronization index among frontal channels during emotional stimulation, particularly during negative emotion (i.e., sadness); furthermore, phase synchronization patterns were found to differ between positive and negative emotions. Costa et al. (2006) also found that sadness was more synchronized than happiness at each frequency band and was associated with a wider synchronization both between the right and left frontal sites and within the left hemisphere; in contrast, happiness was associated with a wider synchronization between the frontal and occipital sites.

Although the aforementioned emotional specificity EEG studies that have used functional connectivity have nicely demonstrated that EEG-based functional connectivity can effectively differentiate among different emotional states, none of these studies have yet directly compared these different connectivity indices in a single study. We believe that this research topic is worth pursuing because different connectivity indices are sensitive to different characteristics of EEG signals. Correlation is sensitive to phase and polarity, but it is independent of amplitudes, and changes in both amplitude and phase lead to a change in coherence [20]; similarly, the phase synchronization index is influenced only by a change in phase [29]. Therefore, the present study aimed to re-address the issue of EEG-based emotional specificity using the three functional connectivity indices and to compare which of the indices are better able to recognize different emotional states based on pattern classification analyses.

Although previous studies have tried to classify emotional states by means of recording and statistically analyzing EEG signals from the central nervous systems [16]–[18], [30]–[32], however most of these pioneer works focused on EEG features extracted at the single electrode level. We believed that connectivity indices such as correlation, coherence, and synchronization index may also provide meaningful information, since processing of emotions is a complex procedure and is not completed solely by one or few specific brain regions.

We used emotional film clips to elicit three different emotional states. These emotional states are based on the dimensional theory of emotion, which asserts that there are neutral, positive, and negative emotional states, because numerous studies have suggested that the responses of the central nervous system [33]–[36] correlate with emotional valence and arousal. More critically, as suggested by Mauss and Robins (2009), “measures of emotional responding appear to be structured along dimensions (e.g., valence, arousal) rather than discrete emotional states (e.g., sadness, fear, anger)” [11]. This study can be considered a pioneering study that investigates emotional specificity in patterns of EEG-based functional connectivity using three different connectivity indices and directly compares the connectivity indices to determine which are the most powerful in correctly recognizing different emotional states.

## Materials and Methods

### 1. Ethics Statement

All subjects signed informed consent before the experiments, which was approved by the Chung Cheng University ethical review committee (IRB).

### 2. Participants

Participants were 40 healthy, right-handed students from National Cheng Kung University (21.43±1.33 yr, 21 males; 21.83±1.70 yr, 19 females). Individuals with a prior history of neurological or psychiatric illness or current or prior psychoactive medication use were excluded. Participants were asked to abstain from caffeine and tobacco use for 24 hours before testing. Each participant was paid NT $1,000 (US $30) for approximately 6 hours of participation.

### 3. Emotion-Eliciting Film Clips

Six emotion-eliciting film clips with visual and auditory components were retrieved from the Standard Chinese Emotional Film Clips Database [37] to induce positive (an amusing & a surprising film clip), neutral (two “neutral” film clips), or negative (a fear & a disgust film clip) emotions. The duration of each film clip ranged from 0.5 to 5 minutes. Previous studies have also used film clips with varied durations as emotional stimuli (see Christie and Friedman, 2004; Gross and Levenson, 1995). We believed that varied durations of the film clips would not influence stimulus processing per se.Each film clip was edited to create a coherent segment and thus maximize the emotional meaning of each clip. The auditory volume was kept constant for each film clip. To avoid order effects on emotion elicitation, the presentation of the six film clips was counterbalanced using a Latin square design.

### 4. EEG Measurement

EEG was recorded with 64-channel Neuroscan equipment (NeuroScan 4.3.1, USA), according to the international 10–20 system. A ground electrode was attached to the center of the forehead. Electrooculography (EOG) was measured to control for ocular artifacts. Vertical eye movement was measured using electrodes placed above and below the left eye, and horizontal eye movement was measured with electrodes placed lateral to the *left and right* external canthi. EEG and EOG signals were amplified using a multichannel biosignal amplifier (band pass 0.1–100 Hz) and A/D converted at 500 Hz per channel with 12-bit resolution. The impedance of each electrode had to be less than 5 kΩ.

### 5. Experimental Procedure

The experiment began with a 60-s go/nogo task to keep participants in a neutral emotional state before watching a film clip. Then, two 90-s baseline resting EEGs were recorded, with the participant’s eyes open in the first and closed in the second. These baselines were followed by the emotional film clips trial. Each clip trial consisted first of a brief countdown to increase attention (from 5 to 1 with a step of 1 count per second), presented on the monitor, followed by the clip presentation, which was approximately 0.5–5 mins long. As participants were unlikely to always manifest an emotional response at a consistent time segment for each flim clip, some studies asked participants to press a button when he thought that he had reached a specific emotional status [38]. Hence, in this study, our participants were likewise asked to press the spacebar once and only once while watching each film clip, whenever they felt their emotion had changed as a result of watching the clip. Finally, each clip trial ended with a 60-s post-film resting period. After watching the film clips, the experimental affect was assessed using the self-assessment manikin (SAM) developed by Lang, which assessed the participants’ valence, arousal, dominance level, using 9-point scales.

### 6. Signal Preprocess and Data Analysis

A 16.384-s (8192 data-points) signal was extracted before the time point at which the participant had clicked on the spacebar to indicate his or her felt emotion. We used 16.384 sec interval (8192 data points, 500 Hz sampling rate) for the following reasons: (1). In order to obtain a stable estimate of spectral power, Davidson suggested that a minimum of approximately a 10 s signal would be required if the dependent measure was an electroencephalogram (EEG) (Davidson, 1990). He also mentioned that brain activity occurring over long period might be interfered with by factors which are unrelated to the elicited emotion. (2). For Coherence estimation, EEG signal was transfer to frequency domain via fast fourier transform (FFT). To increase transform speed, the number of data points should be power of 2. A custom Matlab 2009b (Waltham, MA, USA) program was used for offline data analysis. To remove 60-Hz noise coming from the power line, the EEG signal was passed through a low-pass filter with a 50-Hz cutoff frequency. EEGLAB, an open-source toolbox for analysis of single-trial EEG dynamics, was used in this research [39]. The eye movement component in the raw data was detected and removed using an independent component analysis (ICA) routine in EEGLAB. The artifact remove procedure was applied to all EEG epochs for every subject under different emotion conditions since it is hard for participants to keep eyes fixed during watching entire film clips. The amount of artifact removal was similar across different emotion conditions. A 2nd order bandpass Butterworth filter was used to extract the specific EEG frequency bands. To estimate the EEG-based functional connectivity among brain sites, the following three indices were calculated for the theta band (4–7 Hz), alpha band (8–12 Hz), beta band (13–30 Hz) and gamma band (31–50 Hz).

### 7. Functional Connectivity Indices

Following the suggestion by Costa [28], EEG-based functional connectivity was estimated in the theta, alpha, beta, and gamma bands for all pairs of 19 electrodes, including Fp1, Fp2, F7, F8, F3, F4, Fz, C3, C4, Cz, T7, T8, P7, P8, P3, P4, Pz, O1, and O2.

#### 7.1 Correlation

For two different signals A and B, the correlation at each frequency (f) is defined as
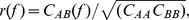
(1)where C_AB_ is the cross-covariance between signals A and B; C_AA_ is the auto-covariance of signal A; and C_BB_ is the auto-covariance of signal B. Correlation is sensitive to phase and polarity and its value ranges from −1 to 1. A higher correlation corresponds to a stronger relationship between two brain sites.

#### 7.2 Coherence

For two different signals A and B, the coherence at each frequency (f) is defined as
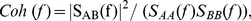
(2)where 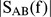 is the cross-spectral density between signals A and B; 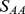 is the auto-correlation of signal A; and 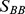 is the auto-correlation of signal B. Coherence is sensitive to amplitude and phase change, and its value ranges from 0 to 1. Similar to correlation, higher coherence indicates that two brain sites are working more closely together, but at a specific frequency.

#### 7.3 Phase synchronization index

Phase synchronization between two nonlinear oscillation systems is defined as
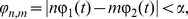


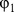
 and 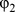 are the phases of two oscillation systems and α is a constant. To compute phase synchronization, it is necessary to obtain the phase of the signal. The instantaneous phase of any signal x(t) is defined as
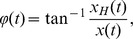
where 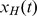 is the Hilbert transform of x(t).

After finding the instantaneous phases of two signals, the phase differences between two signals (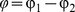) can be obtained by setting m = n = 1. For two signals with data length L, the phase synchronization index (PSI) is defined as
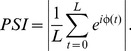
(3)


The phase synchronization index is sensitive to phase change and its value ranges from 0 to 1. The phase synchronization index = 1 if and only if the condition of strict phase locking is obeyed. In contrast, the phase synchronization index = 0 for uniformly distributed phases.

### 8. Statistical Analysis

To determine the different connectivity indices among all pairs of 19 electrodes, repeated-measures analyses of variance (ANOVAs) for each frequency band were used, with two within-subject factors: electrode pair (171 pairs) and condition (neutral, positive, and negative emotion). Post-hoc analyses were calculated using Tukey tests to compare functional connectivity patterns within the specific states of neutral, positive, and negative.

### 9. Using Pattern Classification Analysis to Recognize Emotional States Based on EEG Connectivity Indices

To test whether the pattern of connectivity indices could be used to predict emotional states, the connectivity indices for all pairs of electrodes at each frequency band were selected as features for pattern classification. The term “feature” in pattern classification analysis refers to a psychophysiological variable [40]. To define an optimal set of features, a criterion function should be defined. Thus, an exhaustive search in all possible subsets of input features was required to guarantee an optimal set. To limit this enormous search space, we performed feature selection based on ANOVAs (Kreibig et al., 2007). Features for which ANOVAs were significant at *p≤*0.05 (Tables 1, 2, & 3) were selected, as this led to the best precision. A simple classifier named Quadratic Discriminant Analysis (QDA), contained in the MATLAB Arsenal software [41], was used in this study. QDA is closely related to Linear Discriminant Analysis (LDA). It provides extremely fast evaluations of unknown inputs performed using distance calculations between a new sample and the mean of training data samples in each class, weighted by their covariance matrices. A quadratic discriminant Analysis tries to find an optimal hyperplane to separate the three emotional states (neutral, positive and negative). In this study, 2-fold cross validation was used for pattern classification. For each fold, we randomly assigned data points to two sets as “data 1” and “data 2”, so that both sets of data were of equal size. We first trained on data 1 and tested on data 2, and then trained on data 2 and tested on data 1. This has the advantage that training and test data are both large, and each data point is used for both training and testing on each fold. If the predicted emotion matched the target emotion (in testing data), that indicated the data were correctly classified. Accuracy was defined as the percentage of data which were correctly classified. For example, if there were 100 testing data, and 60 of the data were correctly classified, then the accuracy was 60%. The 2 accuracies from the 2 folds then averaged to produce a single accuracy estimation; However, Brodersen et al. 2010 argued that the average accuracy may lead to false conclusions since a classifier was tested on an imbalanced dataset. To overcome the shortcoming, they proposed the “balanced accuracy” instead of average accuracy [42].

**Table 1:** Significant Results of F-test and Tukey Test of Correlation (p<.05).

θ	FP1-F8	*F* = 3.41	FP1-Cz	*F* = 3.22	FP1-T8	*F = *5.42	FP1-P7	*F = *3.84	FP2-F7	*F = *5.28
	FP2-F8	*F* = 11.44	FP2-C3	*F* = 4.33	FP2-Cz	*F = *3.42	FP2-T8	*F = *7.69	FP2-P8	*F = *3.50
	F7-F8	*F* = 3.42	F7-F4	*F* = 3.56	F7-P7	*F = *5.82	F7-O1	*F = *3.21	F3-F4	*F = *3.83
	F3-P7	*F* = 4.79	F3-O1	*F* = 3.58	F4-C3	*F = *3.96	F4-T8	*F = *8.73	F4-P8	*F = *3.53
	Fz-T8	*F* = 7.09	C4-P7	*F* = 3.96	T7-T8	*F = *4.29	T8-P7	*F = *7.33	T8-P3	*F = *3.90
	T8-O1	*F* = 7.91	T8-O2	*F* = 5.81	P7-P8	*F = *5.57	P8-O1	*F = *6.02	P8-O2	*F = *4.88
	O1-O2	*F* = 4.12								
α	F7-F8	*F* = 3.18	F7-P7	*F* = 3.45	C3-T8	*F = *3.41	T7-T8	*F = *3.92	T7-P7	*F = *3.19
	P7-Pz	*F* = 3.65	P8-O1	*F* = 4.45	P3-Pz	*F = *4.30	P4-O1	*F = *5.45	Pz-O1	*F = *5.27

**Table 2:** Significant Results of F-test and Tukey Test of Coherence (p<.05).

θ	FP1-P7	*F = *3.93	FP2-F8	*F = *15.01*9*	FP2-C4	*F = *3.86	F7-P8	*F = *3.73	F8-F4	*F = *4.29
	F8-P7	*F = *3.54	F8-O2	*F = *3.44	F3-F4	*F = *5.65	F3-T8	*F = *7.61	F3-P7	*F = *3.85
	F4-P8	*F = *4.57	Fz-T8	*F = *9.00	C3-O1	*F = *4.94	C3-O2	*F = *3.80	C4-T7	*F = *3.39
	C4-P7	*F = *3.38	T8-O2	*F = *3.78	P7-P8	*F = *4.40	P8-O1	*F = *6.25	P8-O2	*F = *5.62
α	FP2-F3	*F = *3.33	F4-P8	*F = *3.74	C4-Cz	*F = *3.46	Cz-T8	*F = *3.72	T7-O1	*F = *3.86
	T8-O2	*F = *4.25	P7-P4	*F = *3.50	P8-O1	*F = *5.07	P3-Pz	*F = *3.80	O1-O2	*F = *3.55
β	F7-P7	*F = *4.52	F7-P8	*F = *3.44	T7-P8	*F = *6.57	P7-P4	*F = *3.59		

Base on the confusion matrix.

Actual (+)Actual (−)

Predicted (+)TPFP

Predicted (−)FNTN

The balanced accuracy which was used in this study can be defined as
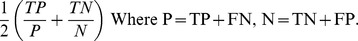
Thus, the discriminability of the emotional states, based upon respective input features, was tested against chance.

In this study, we also compared classification performance using features extracted at the single electrode level to that derived from EEG-based functional connectivity in pairs of electrodes. We followed two methods of feature extraction: one proposed by Dan [15], which is carried out using the Fourier transform. In their study, Fast Fourier Transform (FFT) with a 1 s non-overlapping window was used to compute the energy of each channel and frequency band. Then the log energy was calculated for each 1 s EEG epoch as classification features. Another method was proposed by Murugappan [17], which is based on wavelet analysis. The raw EEG signals were decomposed into sub frequency bands by using Discrete Wavelet Transform (DWT). “db4” wavelet function was used for deriving a set of conventional and modified energy based features for classification. However, we extracted features from 19 electrodes used for EEG-based functional connectivity estimation. These electrodes are not exactly the same as those used in previous studies. Therefore, the classification performance used in our study might not be the same as that proposed in these two studies.

## Results

### 1. Data Preprocessing: Participants Screening

To verify if a film clip successfully elicited the targeted emotional valence states, participants SAM score for each film clip was assessed. We used the following criteria to screen participants based on the valence scores they reported on the SAM. A negative emotional state was defined as low in valence (less than 3), a positive emotional state was defined as high in valence (more than 7), and a neutral emotional state was defined as a valence score between 4 and 6. Only data from participants, whose three targeted emotional states were all successfully elicited by the film clips, were used for further analysis (29 out of 40 individuals). Using this screening procedure, twenty-nine out of the forty participants were selected.

Because the number of film clips for each emotional state that could successfully induce these twenty-nine participants’ targeted emotional states could vary, i.e., some participants had a positive emotional state successfully induced by only one film clip, whereas others had such a state induced by two film clips, our next step was to select one of the two film clips for each targeted emotional state based on these 29 participants’ SAM arousal scores. The data for the positive and negative emotional states with the highest arousal scores were selected as target emotion data for further analysis, and the data for neutral emotional states with the lowest arousal scores were selected. Following this selection procedure, 16 participants had positive emotional states induced by an amusing film clip, 13 participants had positive states induced by a surprising film clip, 13 participants had negative emotional states induced by a fear-related film clip, 16 participants had negative states induced by a disgust-related film clip, 20 participants had a neutral emotional state induced by one of the “no emotion” film clips, and 9 participants had a neutral state induced by the other “no emotion” film clip.

### 2. EEG-Based Functional Connectivity Indices

As Tables 1, 2, and 3 show, the main effect for emotional state was statistically significant for a number of different electrode pairs. Post-hoc analyses were performed using Tukey tests to compare connection patterns under the specific emotional states of neutral, positive, and negative. Since Tukey tests were used in this analysis, multiple comparisons correction was not necessary here.

**Table 3:** Significant Results of F-test and Tukey Test of Phase Synchronization Index (p<.05).

θ	FP1-Cz	*F* = 3.25	FP2-P3	*F* = 4.22	FP2-Pz	*F* = 4.88	F4-T8	*F* = 5.28	C3-C4	*F* = 3.56
	C4-P3	*F* = 4.21	Cz-Pz	*F* = 3.38	T7-Pz	*F* = 6.08	T8-P7	*F* = 4.46	P7-P8	*F* = 3.66
	P7-O2	*F* = 4.80								
α	FP2-F7	*F* = 4.22	FP2-P3	*F* = 3.47	F7-Cz	*F* = 6.47	F7-P7	*F* = 4.93	F3-F4	*F* = 4.49
	F4-T8	*F* = 3.85	Fz-C4	*F* = 4.09	Fz-P4	*F* = 4.94	C3-C4	*F* = 3.45	C3-P3	*F* = 3.39
	C3-P4	*F* = 3.57	T7-O1	*F* = 3.31	P8-O1	*F* = 3.28				
β	FP1-F7	*F* = 3.64	FP2-T8	*F* = 3.54	FP2-Pz	*F* = 3.26	F7-O2	*F* = 3.28	F8-Fz	*F* = 5.97
	F8-P8	*F* = 4.57	F3-Fz	*F* = 5.73	Fz-P4	*F* = 4.36				
γ	FP1-P7	*F* = 3.97	FP1-P4	*F* = 3.24	FP2-Fz	*F* = 4.85	FP2-T7	*F* = 4.68	FP2-T8	*F* = 3.91
	FP2-Pz	*F* = 3.53	F8-P7	*F* = 3.39	C3-C4	*F* = 4.07	Cz-Pz	*F* = 4.84	Cz-O2	*F* = 3.27
	T8-Pz	*F* = 3.58	P3-P4	*F = *3.39						

**Figure 1:**
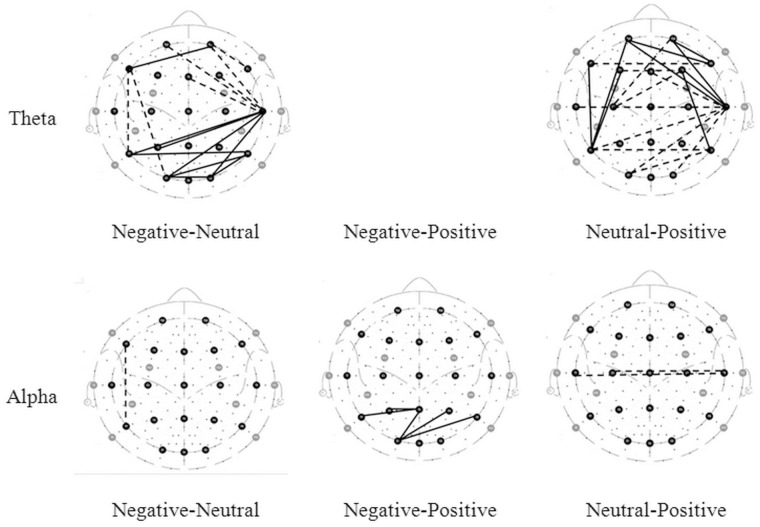
Brain maps of correlation. The lines connecting electrode sites indicate significant higher (solid) and lower (dashed) of correlation values for condition listed on left site.

**Figure 2:**
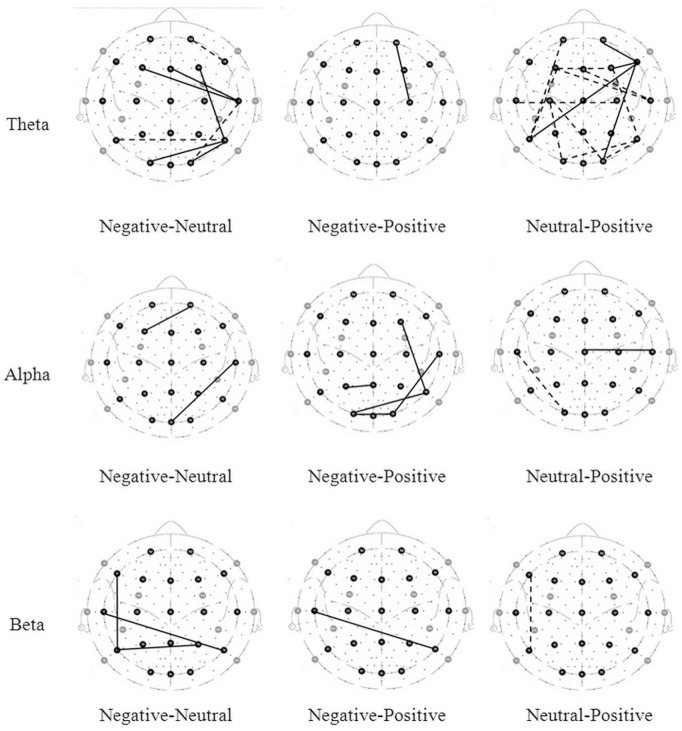
Brain maps of coherence. The lines connecting electrode sites indicate significant higher (solid) and lower (dashed) of coherence values for condition listed on left site.

**Figure 3:**
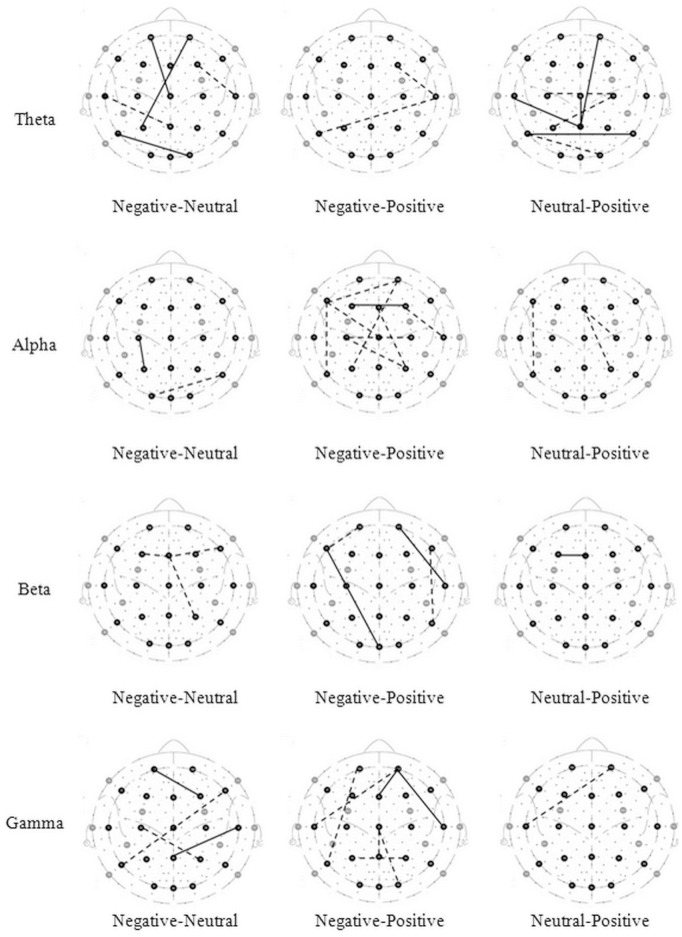
Brain maps of phase synchronization index. The lines connecting electrode sites indicate significant higher (solid) and lower (dashed) of phase synchronization index for condition listed on left site.

The connections with significant difference tested of three connectivity indices value are shown in Figures 1, 2, and 3 (the solid lines connecting electrode sites indicate the index that have significant higher and dashed lines a lower values for condition listed on left site). The details are described below.

#### 2.1 Correlation

The correlation analysis of emotional states is shown in Table 1. The F-test of these correlations produced significant results, mainly in the theta and alpha bands. Significant post-hoc analyses for each frequency band between emotional states are shown in Figure 1 and described below.

1. **Theta band.** Compared to neutral emotions, a significantly lower correlation at the frontal site and higher correlations at the temporal and occipital sites were found when watching negative films. No differences between a negative state and a positive state were found in the theta band. A significantly lower correlation was found in a positive state than in a neutral state at the frontal and parietal sites. A positive state showed higher correlations than a neutral state mainly at the temporal, parietal and occipital sites.
2. **Alpha band.** A significantly higher correlation was found in a neutral state only in the case of F7-P7 activity. A negative state showed a significantly higher correlation than a positive state, especially at the parietal and occipital sites. A neutral state showed a lower correlation than a positive state mainly at the right temporal site.
3. **Beta band.** No significant difference in correlation was observed among emotional states in the beta band.
4. **Gamma band.** No significant difference in correlation was observed among emotional states in the gamma band.

#### 2.2 Coherence

The coherence analysis of emotional states is shown in Table 2. The F-test of these correlations produced significant results, mainly in the theta, alpha and beta bands. Significant post-hoc analyses for each frequency band between emotional states are shown in Figure 2 and described below.

1. **Theta band.** Compared to a neutral state, there was significantly higher coherence at the frontal and right parietal sites when watching a negative film. Compared to a positive state, significantly higher coherence was found during a negative state only for FP2-C4 electrodes. A positive state showed significantly higher coherence than a neutral state, mainly at the frontal and temporal sites.
2. **Alpha band.** A negative state showed significantly higher coherence than a neutral state in the case of FP2-F3 and T8-O2 activity. Compared to when watching a positive film, significantly higher coherence was observed mainly at the temporal and occipital sites when watching a negative film. Compared to a positive state, a neutral state showed significantly higher coherence in the case of Cz-T8 activity and significantly lower coherence in the case of T7-O1 activity.
3. **Beta band.** A negative state showed significantly higher coherence than a neutral state, mainly at the temporal site. A negative state showed significantly higher coherence than a positive state only in the case of T7-P8 activity. A neutral state showed significantly lower coherence than a positive state in the case of F7-P7 activity.
4. **Gamma band.** No significant differences were observed among emotional states in the gamma bands.

#### 2.3 Phase synchronization index

The statistical analysis of the phase synchronization index for emotional states is shown in Table 3. The F-test of these correlations produced significant results at each frequency band. Significant post-hoc analyses for each frequency band between emotional states are shown in Figure 3 and described below.

1. **Theta band**. A negative state was associated with more synchronization than a neutral state, mainly at the frontal site. A positive state was associated with more synchronization than a negative state in the case of F4-T8 and T8-P7 activity, which are associated with temporal sites. A neutral state was associated with more synchronization at the temporal and parietal sites, compared to a positive state.
2. **Alpha band.** A negative state was associated with more synchronization than a neutral state in the case of C3-P3 activity and less synchronization in the case of P8-O1 activity. Compared to both a negative and a neutral state, a positive state showed more synchronization, mainly at the frontal site.
3. **Beta band.** A neutral state was associated with more synchronization than a negative state, mainly at the frontal site. A negative state was associated with more synchronization at frontal and temporal sites and less synchronization at the temporal site. A neutral state showed higher synchronization than a positive state in the case of F3-Fz activity.
4. **Gamma band.** Compared to a neutral state, a negative state showed more synchronization in the case of Fp1-P4 and T8-Pz activity and less synchronization in the case of F8-P7 and C3-C4 activity. A positive state showed more synchronization than a negative state at the frontal and parietal sites. A neutral state showed less synchronization than a positive state only in the case of FP2-T7 activity.

### 3. Pattern Classification Analysis Based on EEG Connectivity

Classification performance was estimated by calculating accuracy, which is defined as the correspondence between the classification result and the input (i.e., when the predicted emotion state matches the actual emotion state). Paired *t*-test was used to assess the significance of the accuracies.

Tables 4, 5, 6 show the mean classification accuracy across 50 trials, based on different connectivity indices with and without feature selection. Classification accuracy based on correlation is shown in Table 4. The results indicate that this classification accuracy was significantly improved with feature selection (in the theta (*t*(49) = 14.64, *p*<.01) and alpha (*t*(49) = 17.61, *p*<.01) bands) and was significantly better than chance in the theta and alpha bands. Classification accuracy based on coherence is shown in Table 5. With feature selection, classification accuracy was significantly improved (in the theta (*t*(49) = 16.01, *p*<.01), alpha (*t*(49) = 18.02, *p*<.01), and beta (*t*(49) = 26.52, *p*<.01) bands) and was significantly better than chance in the theta, alpha and beta bands. Classification accuracy based on the phase synchronization index is shown in Table 6. This classification accuracy was significantly improved with feature selection (in theta (*t*(49) = 16.67, *p*<.01), alpha (*t*(49) = 13.90, *p*<.01), beta (*t*(49) = 17.64, *p*<.01, and gamma (*t*(49) = 18.92, *p*<.01) bands) and was significantly better than chance at all frequency bands. We used the connectivity indices for all frequency bands as features; the results are shown in Table 7. Classification accuracy was significantly improved with feature selection (for correlation (*t*(49) = 27.36, *p*<.01), coherence (*t*(49) = 27.09, *p*<.01), and the phase synchronization index (*t*(49) = 28.45, *p*<.01)). Furthermore, classification performance based on all frequency bands was significantly better than classification performance based on any single frequency band (Table 8). *Bonferroni correction* was used for this multiple comparison. (Statistically significant p-value after the *Bonferroni correction* for correlation comparison: 0.025; for coherence comparison: 0.016; for PSI comparison: 0.0125).

**Table 4:** Comparison of Classification Accuracy in Different Frequency Bands With and Without Feature Selection Base on Correlation.

Correlation	Without feature selection	With Feature selection	
	Mean (S.D.)	Mean (S.D.)	*p*
Theta	0.47 (0.03)	0.55 (0.02)**	1.26×10^−25^
Alpha	0.42 (0.03)	0.53 (0.02)**	5.79×10^−32^
Beta	0.40 (0.02)	NS	
Gamma	0.40 (0.02)	NS	

*Note:* NS: No couples of electrodes were selected since there was no main effect among emotional states (i.e. no feature selection was performed);

**Accuracy with feature selection is significant higher than that without feature selection, *p*<.01.

**Table 5:** Comparison of Classification Accuracy in Different Frequency Bands With and Without Feature Selection Base on Coherence.

Coherence	Without feature selection	With Feature selection	
	Mean (S.D.)	Mean (S.D.)	*p*
Theta	0.46 (0.03)	0.55 (0.03)**	7.38×10^−29^
Alpha	0.44 (0.03)	0.54 (0.03)**	4.15×10^−32^
Beta	0.40 (0.02)	0.53 (0.04)**	1.64×10^−46^
Gamma	0.40 (0.04)	NS	

*Note:* NS: No couples of electrodes were selected since there was no main effect among emotional states (i.e. no feature selection was performed);

**Accuracy with feature selection is significant higher than that without feature selection, *p*<.01.

**Table 6:** Comparison of Classification Accuracy in Different Frequency Bands With and Without Feature Selection Base on Phase Synchronization Index (PSI).

PSI	Without feature selection	With Feature selection	
	Mean (S.D.)	Mean (S.D.)	*p*
Theta	0.53 (0.05)	0.69 (0.05)**	2.33×10^−30^
Alpha	0.50 (0.05)	0.64 (0.05) **	9.49×10^−25^
Beta	0.48 (0.04)	0.65 (0.05)**	1.05×10^−31^
Gamma	0.52 (0.06)	0.70 (0.04) **	5.33×10^−32^

*Note:* **Accuracy with feature selection is significant higher than that without feature selection, *p*<.01.

**Table 7:** Comparison of Classification Accuracy by Using All Frequency Bands Base on Correlation, Coherence and Phase Synchronization Index (PSI) With and Without Feature Selection.

	Without feature selection	With feature selection	
	Mean (S.D.)	Mean (S.D.)	*p*
Correlation	0.43(0.04)	0.61 (0.03)**	2.22×10^−47^
Coherence	0.44 (0.03)	0.62 (0.04) **	3.32×10^−47^
PSI	0.52 (0.06)	0.82 (0.06) **	9.48×10^−38^

*Note:* **Accuracy with feature selection is significant higher than that without feature selection, *p*<.01.

**Table 8:** Comparison of Classification Accuracy by Using Single Frequency Band Against Using Total Frequency Bands Base on Correlation, Coherence and Phase Synchronization Index with Feature Selection.

	Total -Theta			Total -Alpha			Total - Beta			Total - Gamma		
	*df*	*t*	*p*	*df*	*t*	*p*	*df*	*t*	*p*	*df*	*t*	*p*
Correlation	49	10.37**	4.84×10^−17^	49	14.65**	2.76×10^−26^	–			–		
Coherence	49	11.77**	2.41×10^−20^	49	14.05**	2.26×10^−24^	49	16.19**	2.80×10^−28^	–		
PSI	49	17.11**	1.18×10^−27^	49	24.08**	2.34×10^−37^	49	21.27**	1.19×10^−32^	49	18.78**	2.20×10^−32^

*Note:* **Accuracy by using total frequency band is significant higher than that using single frequency band, *p*<.01.

To determine which index for emotion classification was best, we compared classification performance using the connectivity indices for all frequency bands, with feature selection; the result is shown in Table 9. Classification accuracies were analyzed using repeated-measures ANOVAs; if these analyses were significant, post-hoc Tukey tests were performed. The mean accuracy was 0.61 for correlation, 0.62 for coherence and 0.82 for the synchronization index, separately. The results of repeated-measures ANOVAs showed that significant main effects were present. Post-hoc Tukey tests indicated that the prediction accuracy of the phase synchronization index was significantly higher than that of the correlation and coherence analyses (*F(2,49) = *12492.56, *p*<.01).

**Table 9:** Comparison of Classification Accuracy by Different Connectivity Index.

		ANOVA	Tukey HSD test
Connectivity index	Mean (S.D.)	F	Correlation	Coherence	PSI
Correlation	0.61 (0.03)				
Coherence	0.62 (0.04)	12492.56**		> Correlation**	
				(*p* = 0.03)	
PSI	0.82 (0.06)				> Correlation**
					(*p* = 1.52×10^−57^)
					> Coherence**
					(*p* = 3.22×10^−51^)

df = 2/49.

*Note:* *p<.05. **p<.01.

## Discussion

The goal of this study was to demonstrate EEG-based functional connectivity among different emotional states, which was investigated by estimating the dynamic coupling between EEG channels associated with emotion. This study is the first to investigate brain functional connectivity among emotional states using three different indices. The main prediction, i.e., that different patterns of functional connectivity would be associated with different emotional states, was supported by our results. Furthermore, differences in functional connectivity can be considered a feature, and this feature can be used to predict emotional states.

One might argue that the EEG pattern was elicited by the specific features of film clips (i.e. image, sound…), not emotion per se. However, we think this is unlikely for two reasons. First, in our study, we found that even the same movie clip might induce different emotions for different individuals, which led to the emotional valence did not match the targeted emotion for some individuals (15% in positive emotion, 9% in neutral emotion and 29% in negative emotion). To overcome this problem, in our study, the experimental affect was assessed using the self-assessment manikin (SAM). Only data from participants whose two targeted emotional states were all successfully elicited by the film clips were used for further analysis.

Second, two different films for each emotion were used which are positive (one amusing & one surprising film clip), neutral (two “neutral” film clips), and negative (one fear & one disgust film clip) emotions. Two sets of clips (two clips for each target emotion) were usually used in emotional research which allowed us to test whether EEG reactions were specific to the emotion domain represented by the film clips or to specific aspect of the film clips. If similar EEG patterns were observed in two different film clips for single target emotion, we can conclude that the EEG pattern difference was due to emotion but not some basic aspect of the film clips.

### 1. EEG-Based Functional Connectivity among Scalp Regions

As observed in Tables 1 to 3 and Figures 1 to 3, different EEG-based functional connectivity patterns were observed among different emotional states. Shin and Park (2011) proposed that correlation coefficients between the channels in temporal and occipital scalp regions would increase with negative emotion [25]. Similar results were found in this study; for the theta and alpha bands, a negative emotional state was associated with higher correlations in the occipital site than were neutral and positive emotional states. The current results also showed that a positive emotional state was associated with higher correlations in the temporal site, especially in the right hemisphere, than was a neutral state.

Some studies have reported that coherence was greater during negative emotion than during positive emotion [27]. Similar observations have been reported for prefrontal–temporal EEG coherence while participants were watching stressful versus enjoyable film sequences [43]. In line with these findings, our results showed that coherence was greater during negative emotion than during positive emotion in the theta, alpha and beta bands. Most of these coherence differences were located in the right parietal and occipital sites.

Aftanas et al. used Kolmogorov entropy as a measure and found that positive emotion was associated with a smaller degree of entropy, which indicated more synchronization, especially in frontal sites [44]. Our results similarly indicated that positive emotion was more synchronized than negative emotion at each frequency band. However, in Costa et al.’s (2006) study, the researchers proposed that sadness was more synchronized than happiness at each frequency band. This inconsistency might occur because the emotional stimuli used in the current study were different from those in Costa et al.’s (2006) study. Specifically, we used fear and disgust films as stimuli for negative emotion, which is different from Costa et al.’s study. Kreibig has indicated that sadness and fear might be expected to differ physiologically [40]; hence, different emotions might cause divergent brain synchronization even when both are considered to be negative emotions.

Taken as a whole, the results of the present study agreed with the findings in previous studies that have highlighted the role of integration of brain information in emotional processing. Correlation and coherence analyses showed similar patterns, in that negative emotional states had higher correlation (or coherence) than positive emotional states, especially at the occipital and temporal sites. This is not surprising, considering that Guevara and Corsi-Cabrera’s (1996) research reveals that correlation and coherence show high degrees of statistical equivalence in EEG analysis [20]. However, the phase synchronization index during negative states was smaller than it was during positive states at the frontal site. These divergent findings might be due to differences in the essence of each of these three connectivity indices: they are sensitive to different characteristics of the EEG signal (i.e., amplitude, phase and polarity).

### 2. Pattern Classification Analysis Based on EEG Connectivity

Tables 1 to 3 demonstrate that EEG-based functional connectivity indices show different patterns among emotions. To address whether a pattern can be used as a feature to predict emotional states, QDA classification was used in this research. The results showed that emotional states could be accurately predicted. Classification accuracy was significantly better than chance when using feature selection, which indicates that emotional states might be characterized by unique patterns of EEG-based functional connectivity indices.

Some studies have proposed that appropriate feature selection is essential for achieving good performance. By reducing the number of feature vectors, a more compact and more easily interpretable set of data is provided to the system, the performance of the learning algorithm is improved and the speed of the system increases [45], [46]. Tables 4 to 6 show that classification without feature selection resulted in worse performance than classification with feature selection, indicating that feature selection is relevant for emotion recognition. In this study, we performed the feature selection approach using all data (including training and testing data). However, this might introduce a bias in the final accuracy estimation. In order to preclude the possible bias, we re-analyzed the data with the procedure that the feature selection process was only applied to the training data and not to the testing data for each fold of the cross validation. The results are shown in Table 10. These results are similar to the results reported in this paper, i.e., the classification accuracy was significantly improved with feature selection. Hence we believe that the improvement in classification performance was due to feature selection.

**Table 10:** Classification Accuracy when Feature Selection Process was Only Applied to The Training Data.

	Without feature selection	With feature selection	
	Mean (S.D.)	Mean (S.D.)	*p*
Correlation	0.42 (0.05)	0.54 (0.03)**	1.04×10^−19^
Coherence	0.40 (0.03)	0.61 (0.05)**	1.44×10^−31^
PSI	0.44 (0.04)	0.68 (0.04)**	1.86×10^−30^

*Note:* **Accuracy with feature selection is significant higher than that without feature selection, *p*<.01.

The results of this study demonstrate that connectivity indices at each frequency band can seemingly be used to distinguish different emotional states. Although changes in alpha band activity between different emotions have been well documented [47], it has been proposed that activity in the theta [14], [48]–[53], beta [14], [54] and gamma [55]–[58] bands could also be associated with emotional states. Our data show that EEG-based functional connectivity at each frequency band reveals specific patterns for different emotional states (Tables 4 to 6). Thus the connection between emotions and EEG patterns does not occur in only one particular band, but it is evident in all frequency bands. Several studies have tried to use features extracted from all frequency bands to predict human emotional states [16]–[18], but these studies did not compare classification performance when only features within a single, specific frequency band were used. Tables 7 and 8 demonstrate that if we consider features from all frequency bands, better classification performance is achieved than when only one frequency band is considered. Dan and colleague (2011) proposed that classification performance using all frequency bands was better than that based on individual frequency bands under the same condition [15], and similar results were found in our study. This finding points to the important conclusion that all frequency bands need to be considered in emotion research, rather than restrict an analysis to one particular frequency band.

Table 9 shows that classification based on the phase synchronization index was significantly more accurate than classification based on correlation and coherence. Many studies have attempted to directly study brain interaction by measuring coherence. However, some researchers have asserted that coherence is only suitable for stationary signals because it is a measure of the linear co-variance between two spectra in the frequency domain [29], [59], [60]. Classical methods of spectral estimation were based on the Fourier transform. To estimate coherence, data were subdivided into several segments and were then transferred to the frequency domain. This method requires that each segment have the same spectral properties because the assumption of stationary is not easy to attain in EEG research. Another point, made by Guevara and Corsi-Cabrera (1996), is that changes in both the amplitude and the phase lead to changes in coherence; the relative importance of amplitude and phase change in the coherence value is thus not clear. Similarly, correlation is sensitive to both phase and polarity [20]; hence, the same controversy has existed in correlation estimation. In contrast, the phase synchronization index is influenced only by the change of phase and therefore reveals clearer information about brain interaction; thus, classification performance is better when the phase synchronization index is used.

### 3. Classification Performance Based on Connectivity versus Single-Electrode-Level Measurement

In our study, features from these 19 electrodes were extracted via two methods. The classification accuracy by features extracted base on EEG power was 0.53(0.04) and by features extracted base on wavelet analysis was 0.48(0.06). The results, shown in Table 11, indicate that classification performance was better when using features extracted from EEG-based functional connectivity in pairs of electrodes with feature selection, compared to using single electrodes; thus, EEG-based functional connectivity seems to provide an interesting and useful tool for studying and understanding the mechanisms underlying emotional processing.

**Table 11:** Classification Accuracy by Features Extracted from Signal Electrode.

Feature extraction method	Mean(S.D.)
Features base on power (Dan et al., 2011)	0.53 (0.04)
Features base on wavelet analysis (Murugappan et al., 2010)	0.48 (0.06)

### 4. The Gender Difference in Classification Performance

Some research had suggested that the sex differences in reactivity patterns to emotional stimuli in psychophysiological measures [61], [62]. In our study, although the function connectivity patterns might be different, EEG-based functional connectivity reveals similar effect on classification for different emotional states in either male or female groups. Table 12 shows that classification performance based on correlation and coherence are similar between male and female groups. However, better classification performance was observed in female than male group base on phase synchronization index (*t*(49) = 4.90, *p*<.01 ). Some studies demonstrated that women tend to show hypersensitivity to emotional stimuli [62]. Furthermore, according to Table 9, phase synchronization index seems to be more sensitive to emotions. These might be the reason why better classification performance in female group base on phase synchronization index. Whether the functional connectivity shows different pattern between male and female groups and the underlying mechanism might be an interesting topic in the future.

**Table 12:** Comparison of Classification Accuracy by Using All Frequency Bands Base on Correlation, Coherence and Phase Synchronization Index (PSI) between male and female.

	Correlation	Coherence	PSI
Male	0.61(0.05)	0.65(0.05)	0.75(0.04)
Female	0.57(0.08)	0.64(0.06)	0.79(0.06)*

*Note:* *Accuracy in female is significant higher than that in male, *p*<.05.

### 5. Recommendations for Further Research

This study provided evidences which imply that brain activity is associated with emotional states by estimating three functional connectivity indices. Nonetheless, the present work presented a number of limits and more studies will be needed. First, this study use film clips for emotion induction, however, some experimental manipulations utilized picture viewing [63], facial expression [5], music [14] for emotion induction. Some studies indicated that different kinds of stimuli led to different brain activity [64]. Further studies of EEG-based functional connectivity for different kinds of stimuli would be necessary to build a better understanding of relationship between brain activity and emotion. Second, although correlation, coherence and synchronization index had been used in emotional research, other multivariate methods such as Partial Directed Coherence (PDC) and Directed Transfer Function (DTF) [65] were commonly used to estimate the brain functional connectivity. In the future, further studies of whether such methods can be used as indices for emotion recognization will be needed.

## Conclusion

In summary, our data demonstrate that EEG-based functional connectivity reveals different patterns for different emotional states, in either single or combined frequency bands. This finding provides evidence that emotional states are characterized by their own individual patterns of central nervous system response. Considering both the complex procedure involved in processing emotion and that emotion involves several neural systems at different brain sites, we can conclude that research using EEG-based functional connectivity among brain sites might be a fruitful direction for future research on emotions.
